# Squamous cell cancer of the temporal bone: a review of the literature

**DOI:** 10.1007/s00405-020-06281-4

**Published:** 2020-08-31

**Authors:** Matt Lechner, Liam Sutton, Charlotte Murkin, Liam Masterson, Paul O’Flynn, Michael J. Wareing, Taranjit Tatla, Shakeel Saeed

**Affiliations:** 1 Royal London Hospital, Barts Health NHS Trust, London, UK; 2 UCL Cancer Institute, University College London, 72 Huntley Street, London, WC1E 6DD UK; 3 Department of ENT, Adenbrooke’s Hospital, Cambridge, UK; 4 Head and Neck Centre, University College London Hospitals NHS Trust, Euston Road, London, NW1 2PG UK; 5 Department of Otolaryngology–Head and Neck Surgery, Northwick Park Hospital, Harrow Road, Harrow, London, UK; 6 Royal National Throat, Nose and Ear Hospital, University College London Hospitals NHS Trust, 330 Grays Inn Road, London, WC1X 8DA UK; 7 UCL Ear Institute, 332 Gray’s Inn Road, London, UK

## Abstract

**Background and aims:**

Squamous cell carcinoma (SCC) of the temporal bone is a rare malignancy accounting for only 0.2% of head and neck cancers. There is currently no clear consensus on staging or common approach to management. It is the aim of this work to provide the readers with a review of the current literature on this malignancy.

**Methods:**

A literature review was performed identifying 16 case series with patient numbers ranging from 12 to 124. A total of 708 patients were included in this review, 67% presented with advanced disease. 578 cases were managed operatively with lateral temporal bone resection, some underwent local resection alone in early stage disease. In all studies radiation therapy was used as an adjunct to some degree.

**Results:**

More than half of studies reported 100% either 2-, 3- or 5-year survival for T1 and T2 disease with no nodal involvement. Survival correlated with disease stage and in five studies SCC differentiation was found to be a significant prognostic factor. Post-operative radiotherapy was found to improve survival in only one study.

**Conclusions:**

Temporal bone SCC is a readily treatable malignancy in early stage disease, however late stage disease has a poor prognosis. Differentiation of the SCC and stage of disease at presentation appear to have the greatest influence on 5-year survival rates. Further work is required in both the identification of early stage disease and in the treatment of later T3 and T4 lesions.

**Electronic supplementary material:**

The online version of this article (10.1007/s00405-020-06281-4) contains supplementary material, which is available to authorized users.

## Introduction

Squamous cell carcinoma (SCC) of the temporal bone is a rare malignancy, occurring in less than 6 individuals per million each year, accounting for 0.2% of all head and neck cancers [1, 2]. Despite some improvements in outcome over the past several decades, overall 5-year survival remains relatively poor at 36–67% [3, 4]. While metastatic spread is quite rare, these tumours are locally aggressive due to the number of routes through which the cancer can invade [5, 6]. The structure of the temporal bone facilitates spread via microscopic diffusion through bony canals as well as nerve and intraosseous vessels [7]. Furthermore, due to the asymptomatic nature of the disease early on, most patients present at advanced stage, resulting in poor prognosis.

Risk factors for SCC of the temporal bone include chronic suppurative otitis media and previous radiation therapy [8]. As well, some studies have found an association between oncogenic/high-risk HPV (HPV16/18) and middle ear carcinoma [9, 10]. A common pathological pathway for these various risk factors may be that of chronic inflammation causing subsequent metaplasia and malignant transformation.

At present, there is no consensus concerning TNM staging for these tumours by either the American Joint Committee on Cancer (AJCC) or the Union Internationale Contre le Cancer (UICC). Since the early 1990s, the Pittsburgh classification system has been used, with a modification in 2002 (Supplementary Table 1), and has been shown to have a consistent correlation with prognosis [2]. Importantly, both T and MRI are essential for accurate staging.

The current treatment paradigm largely involves lateral temporal bone resection in order to achieve adequate margins, often with the addition of a superficial or total parotidectomy. Early stage disease may or may not be treated with adjuvant radiotherapy. In late stage disease, obtaining clear margins becomes increasingly difficult. In cases of advanced tumour spread, careful consideration must be taken into account regarding post-operative quality of life, as radical surgery is associated with significant morbidity [11].

## Methods

A PubMed literature search was carried out using the search terms ‘squamous cell carcinoma’ and ‘temporal bone’. All returned articles were manually filtered for relevance and only English-language case series from 2006 to present were included in the analysis. The reference sections of articles from the initial search were further assessed for additional, relevant publications. Clinicopathological data were subsequently pulled and tabulated for review.

Studies were excluded that included non-squamous cell carcinoma or those that included temporal bone SCC from a different primary source, e.g. parotid or cutaneous. All studies were retrospective case studies and as such have some inherent bias and are of lower evidence quality than randomised controlled trials. That being said, systematic reviews of case series have been advocated for rare conditions such as this, in which higher quality studies are infeasible [12].

## Results

Following a literature search, 16 case series of patients with temporal bone SCC were identified which examined prognostic indicators, management and survival outcomes of specifically primary temporal bone squamous carcinoma (Supplementary Tables 1, 2).

A total of 708 patients were included with patient numbers ranging from 12 to 124 cases. From this cohort 389 patients presented with confirmed late stage disease (T3, T4). The largest study did not include a breakdown of the patients by stage. Of the patients with known staging, 67% presented with advanced disease.

Follow-up varied substantially from a median of 11 months in the shortest study to 66 months in the longest. Five studies did not provide median figures for follow-up, however, four provided 5-year survival and the remaining study provided a range for months of follow-up.

578 cases were managed operatively. The choice of operation varied with some studies opting for a local resection in early stage disease and others performing a lateral temporal bone resection. One study assessed the outcome difference in cases treated with either en bloc or piecemeal LTBR and found no significant difference. The role of parotidectomy was also controversial with some studies performing it routinely in all cases and others taking a select approach based on imaging.

Radiation therapy was used to some degree in all studies, however this varied from a blanket approach to select cases and from only post-operative radiation to both pre- and post-operative radiation. One study also used intraoperative radiation in select cases [13].

Where reported, survival correlated with disease stage in all studies. Nine studies reported 100% either 2-, 3- or 5-year survival for T1 and T2 disease with no nodal involvement [3, 4, 7, 14–18]. T4 disease had a poor survival with the lowest 5-year survival figures at 14% and highest at 49% [3, 4]. Comparisons between studies were difficult due to differing reporting of staging (some reporting T stages and some reporting overall stage) and due to the variety of treatments utilised.

Postoperative radiotherapy was found to improve survival in only one study. Gidley et al. found that in T2 disease, surgery and radiotherapy had a 58% 10-year survival while those treated by surgery alone had a 0% survival [1]. One further study showed a trend towards improved outcomes with postoperative radiotherapy and it was felt that the statistical power was limited by patient numbers [6]. Seven other studies found no significant improvement of outcomes with postoperative radiation and three studies did not document it as a variable [7, 13, 14, 16–19]. Leong et al. treated all patients with radiotherapy and as thus improved outcomes could not be assessed [15].

Nine studies examined patient prognosis and node positivity. Masterson, Zanoletti and Lobo et al. all showed a worse outcome with nodal disease [7, 16, 20]. The remaining six studies showed no significant impact [1, 4, 13–15]. It must be said that the majority of patients with advanced stage disease require some form of reconstruction after lateral temporal bone resection. Often, a neck dissection will be done at this stage not just for tissue sampling, but also for optimal vascular anastomosis, e.g. external carotid artery.

Tumour differentiation was found to be a significant factor in patient outcomes in five studies and found to be not significant in two [3, 7, 14, 15, 20]. The remaining nine studies did not include tumour histology in their analysis.

Parotid infiltration was only shown to impact on patient prognosis in the study by Xie et al. [14]. In keeping with the Pittsburgh system (Supplementary Table 1), four of the case series showed a worse prognosis with involvement of the facial nerve [4, 7, 14, 16]. Four studies found that its invasion was not significant with regard to patient prognosis [1, 13, 15]. Three studies determined dural invasion to be a significant prognostic factor whilst five did not. Positive margins were associated with poorer prognosis in three studies.

## Discussion

The warning signs alerting to possible temporal bone SCC are unilateral otorrhoea, especially if serosanguinous and not responding to topical antibiotic treatment. In such a case, the appropriate imaging (usually a combination of CT and MRI for the tumour and ultrasound for the intraparotid and cervical nodal staging), direct inspection and biopsy is required, with discussion of the results and most appropriate management plan at a designated Head and Neck MDT meeting. Outcomes in late stage disease are poor and further multicentre trials are required to establish the optimum management for this patient group.

Our literature review revealed that squamous cell carcinoma of the temporal bone is a readily treatable cancer in early stage disease, however late stage disease has a poor prognosis. Further work is required in both the identification of early stage disease and in the treatment of T3 and T4 lesions. Potential prognostic markers include tumour grade, nodal involvement, positive surgical margins, dural invasion and facial nerve involvement, in addition to tumour stage. On the basis of our review, it seems that the variable that influences survival most is the differentiation of the SCC.

This review suggests that surgery remains the mainstay of treatment, however, there is no one strategy for operative management of temporal bone SCC. The main approaches are en bloc surgical resection ± adjuvant radiotherapy versus radical mastoidectomy/piecemeal resection ± adjuvant radiotherapy.

Potential causes for this include a significant proportion of oncological procedures requiring some piecemeal tumour removal as the main bloc of tissue is already fragmented by the carcinoma. In addition, for some patients the diagnosis is made during mastoid exploration for what seemed to be a routine cholesteatoma/chronic otitis media. In such cases, postoperative radiotherapy may be a pragmatic approach (although this may adversely affect patient survival) due to patient preference.

Regardless of the above, recent case series suggest that the best survival rates are achieved with en bloc extended temporal bone resection and postoperative radiotherapy. This can be demonstrated by analysis of recent results from Zanoletti, Xie, Masterson, Leong et al., which all demonstrate improved survival figures despite treating a cohort of patients dominated by T3 and T4 disease [7, 14, 15, 20].

Tumours which do not invade through the tympanic membrane (T1 and T2) can be removed sufficiently by a lateral temporal bone resection. An extended or subtotal temporal bone resection is required when spread of the malignancy extends medial to the tympanic membrane or into the mastoid (T3/T4 disease). The facial nerve is often sacrificed in this latter procedure and a partial or complete pinna excision may also be necessary. However, the use of a subtotal temporal bone resection may confer a survival advantage in relevant cases, in comparison to lateral resection.

Although nodal disease is uncommon in early stage disease, a neck dissection will provide access to the major vessels for free flap reconstruction and allow accurate staging. A selective or modified radical neck dissection can be performed depending on the presence of macroscopic disease. Node-positive disease is associated with a poor prognosis, and neck dissection does not improve survival.

The distribution of pathological nodes is largely restricted to levels II/III/Va with the exception being level IV in < 10% of cases. On this basis, a selective neck dissection should incorporate this anatomical region when performed for node-negative disease.

## Conclusion

Temporal bone SCC is a readily treatable malignancy in early stage disease, however late stage disease has a poor prognosis. Differentiation of the SCC and stage of disease at presentation appear to have the greatest influence on 5-year survival rates. Future work should focus on the identification of markers of early stage disease and improved treatment modalities for late stage disease. Importantly, collaborative efforts need to be made to overcome the limitations posed by the rarity of this malignancy such that prospective trials of good quality can be conducted. This will be paramount in our ability to better understand the disease and, thus, improve clinical outcome.

## Electronic supplementary material

Below is the link to the electronic supplementary material.

Supplementary material 1 (XLSX 11 kb)
