# Supplementary material for: Squamous cell cancer of the temporal bone: a review of the literature
Source: Eur Arch Otorhinolaryngol. 2020 Aug 31;278(7):2225–8. doi: 10.1007/s00405-020-06281-4 (PMC8165064; doi:10.1007/s00405-020-06281-4)
Supplement: Supplementary file 1 — Supplementary material 1 (XLSX 11 kb) [file 405_2020_6281_MOESM1_ESM.docx]

| **Author** | **Country** | **Year** | **No. cases** | **Follow up**  **(median + range)** | **Survival rates** | **Overall survival rates** | **No.**  **T3-T4^1^** | **Surgery** | **Surgery** | **Neck dissection** | **Parotidectomy** | **Post op radiation** |  |
| --- | --- | --- | --- | --- | --- | --- | --- | --- | --- | --- | --- | --- | --- |
| **Muelleman** | USA | 2018 | 25 | 11 (1-60 months) | Disease-free survival to last follow-up:  60% (piecemeal); 78% (en-bloc) | Mean OS: 38.9 months (en bloc), 37.5 months (piecemeal) | 5 (20%) | Piecemeal LTBR (60%), en-bloc LTBR (40%) | 25 (100%) | ND | ND | 11 (44%) |  |
| **Omura** | Japan | 2017 | 49 | 48 (3-149 months) | 5-year Disease-free survival: 71% | 5 year: 62% | 16 (48%) | LTBR for lesions which did not extend beyond tympanic membrane (34.7%); STBR for those extending to middle ear (32.7%) | 33 (67%) | ND | 5 (10.2%) | 18 (36.7%) |  |
| **Sun** | Hong Kong | 2017 | 30 | 5-245 months | 3-year Disease-Free Survival: 100% (Tis/T1), 75% (T2) | 3-year OS:  T2 or lower – 100%  T3 – 50%  T4 – 31.2% | 19 (63%) | 11 (40.7%) radical mastoidectomy;  10 (37.0%) LTBR;  6 (22.2%) SBTR | 27 (90%) | 2 (6.7%) | 2 (6.7%) | ND |  |
| **Sugimoto** | Japan | 2015 | 12 | 35 (5-86 months) | Cumulative survival rate: 66.7% | 66.7% | 12 (100%) | 2 (16.7%) salvage LTBR following chemoradiotherapy | 2 (16.7%) | ND | ND | ND |  |
| **Masterson** | UK | 2014 | 60 | 14.5 months (1 month-18 years) | 5-year disease-specific survival for the whole  cohort: 44% | 5 year:  44% (37-51%) | 51 (85%) | T2N0M0 = LTBR  Stage 4 = ETBR  T3N0M0 = mainly ETBR but some facial nerve sparing if clear margin on frozen section | 60 (100%) | 60 (100%) | 8 (13.2%) Superficial  52 (86.6%) Total | 40 (66.7%) |  |
| **Xie** | China | 2013 | 39 | 34.9 months  (9-93 months) | 2 year:  Stage I-III = 100%  Stage IV = 22.3% | 2 year:  56.9% | 27 (69.2%) | 3 (7.7%) LR  32 (80.1%) LTBR  1 (2.6%) STBR | 39 (100%) | 7 (17.9%) | 21 (53.8%) Superficial  6 (15.4%) Total | 27 (69.2%) |  |
| **Zanoletti** | Italy | 2014 | 41 | 41 months (1-220 months) | Disease specific survival:  pT1 – 100%  pT2 – 83.3%  pT3 – 75%  pT4 – 38.1% | - | 28 (68.3%) | 2 (4.8%) Partial LTBR  28 (68.3%) LTBR  11 (26.8%) STBR | 41 (100%) | 33 (80.5%) | 37 (90.25) | 23 (56.1%) |  |
| **Leong** | UK | 2013 | 35 | 27 months (3-143 months) | Disease free survival to last follow-up:  Stage I-III = 100%  Stage IV = 41.4% | Disease free survival to last follow-up:  48.6% | 30 (85.7%) | 6 Stage I-III (17.1%) LTBR  29 Stage IV:  25 (71.4%) ETBR  4 (11.4%) LTBR (pre-existing medical conditions) | 35 (100%) | 30 (85.7%) | 7 (20%) | 35 (100%) |  |
| **Bacciu** | Italy | 2013 | 45 | 46.7 months (4.7-88.7 months) | 5 year:  T1 – 100%  T2 – 100%  T3 – 86.2%  T4 – 48.7 % | 5 year:  67.6% | 34 (75.6%) | 21 LTBR (5 T1, 6 T2, 8 T3, 2T4)  24 STBR (7T3, 17 T4) | 45 (100%) | 8 (17.8%) | 8 (17.8%) Superficial  17 (37.8%) Total | 27 (60%) |  |
| **Chi** | China | 2011 | 72 | ND | 5 year:  T1 – 100%  T2 – 66.7%  T3 – 21.1%  T4 – 14.3% | 5 year:  36.2% | 54 (75%) | 8 LR  29 LTBR  35 STBR | 72 (100%) | ND | ND | 66 (91.7%) |  |
| **Gidley** | USA | 2010 | 124 | ND | 5 year:  T1+2 - 48%  T3+4 - 28% | 5 year:  (Incident tumours only 71/124)  38% | ND | 18 Sleeve resection  7 Mastoidectomy  19 LTBR  6 STBR  5 TTBR | 55 (44.4%) | 26 (21%) | 18 (14.5%) Superficial  9 (7.3%) Total | 23 (18.6%) |  |
| **Cristalli** | Italy | 2009 | 17 | 29.5 months (13-73 months) | ND | 3 year:  76.7% | 15 (94.1%) | 14 LTBR  3 ELTBR | 17 (100%) | 17 (100%) | 17 (100%) | 17 (100%) |  |
| **Lobo** | Spain | 2007 | 19 | ND | 5 year:  Stage II – 100%  Stage III – 25%  Stage IV – 16% | 5 year:  37% | 13 (68.4%) | 11 LTBR  1 STBR  5 TTBR | 17 (89.5%) | 6 (31.6%) | 3 (15.8%) Superficial  6 (31.6%) Total | 13 (68.4%) |  |
| **Kunst** | Denmark | 2008 | 28 | 34 months (2-132 months) | 5 year:  T1+2 – 85%  T3+T4 – 46% | 5 year:  64% | 14 (50%) | 12 LR  11 LTBR  2 STBR  3 TTBR | 28 (100%) | 0 | ND | 23 (82.1%) |  |
| **Bibas** | UK | 2008 | 17 | 66 months (28-132 months) | 2 year:  T2 – 100%  T3 – 59%  T4 – 40% | 2 year:  47.1% | 15 (88.2%) | 4 LTBR  11 STP | 15 (88.2%) | 1 (5.9%) | ND | 12 (70.6%) |  |
| **Yin** | Japan | 2006 | 95 | ND | 5 year:  I – 100%  II – 100%  IV – 29.5% | 5 year:  66.8% | 56 (59%) | 6 Simple tumour resection  17 local canal resection  36 partial temporal bone resection  8 STBR | 67 (70.5%) | ND | ND | 35 (36.8%) |  |
| LTBR – lateral temporal bone resection, ETBR - extended lateral temporal bone resection, LR – local resection, STBR – subtotal temporal bone resection, TTBR – total temporal bone resection, STP – subtotal petrosectomy, ND – not documented  1) According to the Pittsburgh classification system | | | | | | | | | | | | | |

**Table 1.** List of twelve case series of patients with temporal bone SCC which examined prognostic indicators, management and survival outcomes of specifically temporal bone squamous cell carcinoma.

| **Author** | **Post op radiation** | **Node +** | **Grade** | **Margin +** | **Dura mater +** | **CN VII +** | **Parotid infiltration** |
| --- | --- | --- | --- | --- | --- | --- | --- |
| **Muelleman** | ND | ND | ND | ND | ND | ND | ND |
| **Omura** | NS | **NS** | **NS** | **+**  **P=0.009** | **+**  **P=0.008** | NS | ND |
| **Sun** | ND | ND | ND | ND | ND | ND | ND |
| **Sugimoto** | ND | ND | ND | ND | ND | ND | ND |
| **Masterson** | Trend  NS P=0.11 | **+**  **P<0.001** | **+**  **P=0.01** | ND | NS | ND | ND |
| **Xie** | NS | NS | **+**  **P=0.007** | ND | NS | **+**  **P=0.000** | **+**  **P=0.000** |
| **Zanoletti** | NS | **+ P=0.012** | **+**  **P=0.005** | NA | **+**  **P=0.001** | **+**  **P=0.049** | ND |
| **Leong** | N/A | NS | **+**  **P<0.05** | ND | ND | NS | NS |
| **Bacciu** | ND | NS | ND | **+ P=0.0042** | **+**  **P<0.0001** | **+ P=0.0002** | NS |
| **Chi** | NS | ND | **+** | **+**  **P<0.05** | ND | ND | ND |
| **Gidley** | **+**  **(T2 disease only)**  **P=0.011** | NS | ND | NS | NS | NS | NS |
| **Cristall** | NS | NS | ND | NA | NS | NS | NS |
| **Lobo** | NS | **+**  **P=O.OO6** | NS | ND | NS | **+**  **P=0.007** | NS |
| **Kunst** | ND | ND | ND | ND | ND | ND | ND |
| **Bibas** | NS | ND | ND | ND | ND | ND | ND |
| **Yin** | NS | ND | ND | **+**  **P<0.0001** | ND | ND | ND |
| NS – not significant, ND – not documented, + – significant effect on prognosis | | | | | | | |

**Table 2:** List of twelve case series of patients with temporal bone SCC and list of factors which were shown to have a significant effect on prognosis
